# Emergence of common concepts, symmetries and conformity in agent groups—an information-theoretic model

**DOI:** 10.1098/rsfs.2023.0006

**Published:** 2023-04-14

**Authors:** Marco Möller, Daniel Polani

**Affiliations:** ^1^ Theory of Complex Systems Group, Institute of Solid State Physics, Technical University of Darmstadt, Germany; ^2^ Adaptive Systems Research Group, Department of Computer Science, University of Hertfordshire, Hatfield, UK

**Keywords:** symmetries, information theory, embodied agents, collective concepts, information bottleneck

## Abstract

The paper studies principles behind structured, especially symmetric, representations through enforced inter-agent conformity. For this, we consider agents in a simple environment who extract individual representations of this environment through an information maximization principle. The representations obtained by different agents differ in general to some extent from each other. This gives rise to ambiguities in how the environment is represented by the different agents. Using a variant of the information bottleneck principle, we extract a ‘common conceptualization’ of the world for this group of agents. It turns out that the common conceptualization appears to capture much higher regularities or symmetries of the environment than the individual representations. We further formalize the notion of identifying symmetries in the environment both with respect to ‘extrinsic’ (birds-eye) operations on the environment as well as with respect to ‘intrinsic’ operations, i.e. subjective operations corresponding to the reconfiguration of the agent’s embodiment. Remarkably, using the latter formalism, one can re-wire an agent to conform to the highly symmetric common conceptualization to a much higher degree than an unrefined agent; and that, without having to re-optimize the agent from scratch. In other words, one can ‘re-educate’ an agent to conform to the de-individualized ‘concept’ of the agent group with comparatively little effort.

## 1. Motivation

In considering plausible models of the environment that should be employed by agents, especially biologically relevant ones, special attention is given to models espousing principles of information parsimony or optimal information processing [1,2]. Notably, this approach de-emphasizes the specification of particular mechanisms in favour of information optimization. As an early example for a concrete model for representation formation from maximum Shannon information processing, we mention the infomax model from [3].

In this vein, we are interested in how agents can model their environment based on informational considerations only. Using a set of infomax principles to do that, one obtains a classification or representation of a given environment (in the following also called concept) for a given agent. Using the perception–action loop from [4], we model the agent and its interaction with the environment, as well as its information-optimal representation formation. Having an information-optimal representation is independent of any downstream tasks relevant for the agent which do not form part of this study.

In general, the representations of the environment developed in such an information maximization process differ from agent to agent. Even in very simple and highly symmetric scenarios, they can considerably vary from agent to agent as a result of the concrete optimization, as different global optima (or local optima with values close to the global optimum) may be found by the optimization process. This observation is more marked in the biological counterpart where individuals also will differ to various extents from each other as to how they represent the environment, even while performing similarly to each other.

One particular issue this raises is how similar the concepts obtained by different agents are when derived from a common environment. In particular, we will study and discuss what the different concepts developed by those agents have in common. Is it possible to develop a concept that is mutually compatible to each of these input concepts (see e.g. [5–7])? If so, what properties of the environment or the agents do such common concepts capture? And how do they relate to the individual agents’ concepts?

We will not model particular mechanisms on how agents agree on a common concept or how they communicate; instead, we will postulate information-theoretical optimality criteria for desirable common concepts. This approach is supported by increasing evidence that approximate information optimality is reproducibly observed as outcome in the evolution of communication [8,9]. Specifically, for our study, we are intentionally ignoring the details of the processes and mechanisms leading to the emergence of concepts. Instead, we focus exclusively on the principles driving the formation of the final concepts and the results they entail as a consequence of seeking informational optimality.

Analysing the quality of concepts with respect to the environments which exhibit significant symmetry, we observed that ‘good’ concepts exhibit more regularities. While this is, to some extent, expected, this led us to analyse the concepts in detail with respect to their symmetries. Specifically, in this paper, we focused on extrinsic and intrinsic symmetries. Extrinsic symmetries consider external (possibly global) operations keeping relevant aspects of the world invariant; intrinsic symmetries do the same for operations that act on the internal perspective of the agents (formally described in section ‘Symmetry’ further below).

In general, concepts emerging from information-maximization principles will, if at all, reflect such symmetries only approximately. We therefore need a measure to characterize to which degree a symmetry is respected by a concept. For this purpose, we will develop an information-theoretical characterization that also covers possibly imperfect, ‘weak’ symmetries.

Even in an agent/environment with clear extrinsic symmetries, concepts extracted by individual agents will in general only reflect these symmetries quite imperfectly, if at all. One hypothesis behind this work was that, once one extracts common concepts from a group of such agents’ individual concepts (as described in section ‘Common concepts’), these might recover the symmetries to a significantly stronger degree. Once this hypothesis is established, we can furthermore ask under which conditions the individual agents can be ‘re-educated’ to identify the more symmetric common concepts directly.

We then proceed to study a second type of symmetry, namely the influence of the agents’ mapping of sensors and actuators to actual changes in the environment; this mapping is identified with the concept of ‘embodiment’ in the parlance of [10]. Based on transformations of this embodiment, we now investigate ‘what the agent considers to be a symmetry of the environment’ from its internal perspective rather than from the perspective of externally given operations.

To achieve the aforementioned goals, we need to find a description that is consistent with a fundamentally information-theoretical picture of the agents and their environment. This includes formulating the development of a common concept of a set of agents and modelling the notion of regularity or symmetry in the language of information theory.

## 2. Background

We will now introduce our model for the agents and their interaction with the world. For this, we first define some notation and quantities. We consider random variables *X*, *Y*, *Z*, …, denoted by capital letters, which take values *x*, *y*, *z*, … in corresponding sets X,Y,Z, … which we assume are finite for the purpose of this paper. For the probability that a given random variable *X* assumes a value x∈X, we write Pr(X=x) or, if it is clear from the context, just *p*(*x*). For the probability for a joint random variable (*X*_1_, …, *X*_*n*_), we will use equivalently both notations $\mathrm{Pr}(X_{1}=x_{1},\;\ldots ,\;X_{n}=x_{n})\equiv$ $p(x_{1},\ldots ,x_{n})$. The (*Shannon*) *entropy* of a random variable *X* is given by

2.1 $$H(X)\;:=-\sum_{x\in X}p(x)\log p(x),$$

whereby the logarithm in this paper is always taken to the basis of 2, and the unit for entropy is given by a *bit*. The *conditional entropy* of *X* given *Y* is given by *H*(*X*|*Y*) := *H*(*X*, *Y*) − *H*(*Y*) and the *mutual information* between *X* and *Y* by

2.2 I(X;Y) :=H(X)+H(Y)−H(X,Y).

We will also use a generalization of mutual information, the *multi-information* of a collection of random variables, *X*_1_, …, *X*_*n*_

2.3 $$I(X_{1};\ldots ;X_{n})\;:=\left[\sum_{i=1}^{n}H(X_{i})\right]-H(X_{1},\ldots ,X_{n}),$$

and its conditional form, when the random variable *Y* is observed

2.4 $$I(X_{1};\ldots ;\;X_{n}|Y)\;:=\left[\sum_{i=1}^{n}H(X_{i}|Y)\right]-H(X_{1},\;\ldots ,X_{n}|Y).$$

To measure the ‘difference’ between two random variables *X*, *Y*, we can use the unnormalized version of the information distance [11]

2.5 D(X,Y) :=H(X|Y)+H(Y|X).

*D*(*X*, *Y*) vanishes precisely when there is a deterministic bijective map between the supports of *X* and *Y*, i.e. when they are *permutation equivalent*. This can be seen from the fact that a vanishing *D*(*X*, *Y*) implies vanishing conditional entropies in both directions, and, for finite sets, this implies a deterministic map defined over the support of the variable conditioned on. By replacing the notion of equality of two random variables by permutation equivalence, and, noting its symmetry and that *D* fulfils the triangle inequality, *D* becomes a metric on the space of random variables.

We model agents in an environment as perception–action loops, for which we will use the formalism from [4] based on *causal Bayesian networks* (CBNs). A CBN is given by a directed acyclic graph G=(N,E) whose nodes n∈N represent random variables *X*_*n*_ and whose edges e∈E⊆N×N represent causal conditional probability dependencies between them. In such a CBN, the distribution of a random variable *X*_*n*_ is given by *p*(*x*_*n*_|*x*_Pa(*n*)_) where $\mathrm{Pa}(n)\;:=\{n^{\prime }\in N|(n^{\prime },n)\in E\}$ is the set of parent nodes *n*′ to node *n*. If a node *n* has no parent nodes, Pa(n)=∅, we identify *p*(*x*_*n*_|*x*_Pa(*n*)_) ≡ *p*(*x*_*n*_) with an unconditional probability distribution. The joint distribution of the whole network is then given by

2.6 $$p(x_{1},\;\ldots ,\;x_{\left|N\right|})=\prod_{n\in N}p(x_{n}|x_{\mathrm{Pa}(n)}).$$

## 3. Model

As a generic model for an *agent* interacting with its surrounding world we use a CBN formulation of the *perception–action loop* [4]. Here, the agent is modelled in discrete time, at each time step *t* *sensing* the *world* *R*_*t*_ through its *sensor* *S*_*t*_ and *manipulating* it through its *actuator* *A*_*t*_, by which the state of the world is changed in the following time step. We assume the probabilistic sensor map *p*(*s*_*t*_|*r*_*t*_), translating a given world state into a sensor signal, and the actuator map *p*(*r*_*t*+1_|*r*_*t*_, *a*_*t*_), causing a world state to change into a new one under the influence of an action, to be given *a priori* and invariant with respect to time *t*. Together, these two maps can be interpreted as forming the *embodiment* of the agent [10].

In addition to that, the model we use permits the agent to store information in a memory state *M*_*t*_ which can later be used to influence actions. The memory is modelled as an abstract, discrete state, with no constraints on how information can be stored, recalled and transformed.

**Figure 1.**
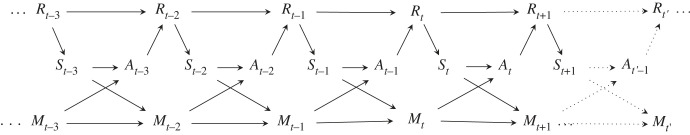
Perception–action loop unrolled in time as a CBN.

This process can be summarized via the CBN shown in figure 1, where we unroll the perception–action loop through time. All random variables are indexed by different time steps *t* : *M*_*t*_, *A*_*t*_, *R*_*t*_, *S*_*t*_.

Apart from the sensing and the actuation, the CBN shows how the agent stores and processes information involving its *memory* *M*_*t*_. This is described by a controller *C*, which, in general, would be realized as a probabilistic mapping *p*(*m*_*t*+1_, *a*_*t*_|*m*_*t*_, *s*_*t*_); however, here we make the more specific assumption that *M*_*t*+1_ and *A*_*t*_ are independent given their parents. This decision is due to the fact that we will limit ourselves to deterministic controllers *C* which will be further justified below. Thus, for the signature of *C*, we will write, by abuse of notation:

3.1 $$C\;:\;M_{t}\times S_{t}\to M_{t+1}\times A_{t}.$$

This controller, like the embodiment maps, is time-independent throughout a run of the agent. Unlike the embodiment maps, however, which are fixed, in the following, the controller *C* will be adapted to optimize certain information-theoretic measures discussed further below. By this, we will model changes of agent behaviour on long timescales to optimize the given measures.

In our experiments, we limited ourselves to consider deterministic controllers *C* because the optimization of information-theoretic measures as used in the present paper typically moves probabilistic controllers *p*(*m*_*t*+1_, *a*_*t*_|*m*_*t*_, *s*_*t*_) towards deterministic mappings. Intuitively, this is due to the fact that in our study we will typically consider the maximization of information uptake; any inclusion of stochastic components will reduce that information and such stochasticity will therefore be suppressed during optimization (for further discussion, see [4,12]). The assumption of a deterministic controller serves to significantly reduce the search space.

The concrete set-up of our experiments consists of a two-dimensional infinite grid-world $R=Z^{2}$. The memory *M* is modelled as a discrete, finite set, concretely, the random variable takes on values in {0,1,2…,|M|−1}. In the experiments, the memory size will be selected to be quite small.

We consider fixed-time runs of the agent in the given world, starting at time *t* = 0. The initial memory *M*_0_ is deterministically set to the default state 0. The random variable denoting the initial position in the world *R*_0_ is uniformly distributed over possible starting positions $R_{0}={\left\{-d,\ldots ,d\right\}}^{2}$ where the *radius* *d* depends on the experiment. The actuator *A* can take on values A={↓,←,↑,→} where these four actions can move the agent (changing its position in the world, encoded in *R*) to one of its four adjacent positions in the grid-world.

**Figure 2.**
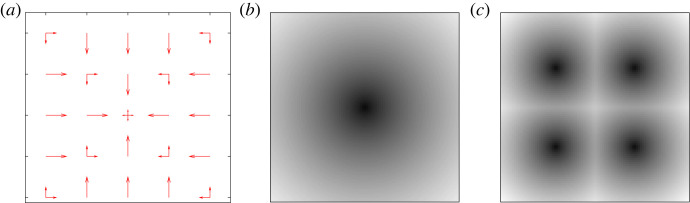
Set-ups.

The first discussed sensor (*set-up s+*) has, similarly, four possible sensor values S={↓,←,↑,→}. Imagine now a ‘pheromone’ gradient emitted by a source at the origin (figure 2*b*). The sensor *S* points to the position adjacent to the agent which contains the highest concentration of pheromone. If the direction of maximum pheromone concentration is not unique (e.g. at the origin), one of these directions is randomly chosen. The set-up s+ is visualized on the left of figure 2, whereby for each position (x,y)∈R all possible sensor ‘directions’ are shown, with their arrow-length corresponding to their probability of occurring. A variation of this set-up used in this work will be *set-up sq*, where, instead of one, four such sources exist at locations {− 5, 5}^2^ (figure 2*c*) and the sensor is always pointing to the nearest source.

Given these set-ups, the fundamental task for the agent will be to capture as much Shannon information about its initial position *R*_0_ as possible in its ‘final’ memory state at time^1^ *t* = 15; this problem class was suggested in [4]. Information-theoretically, this is denoted as maximizing

3.2 $$I(R_{0};M_{15}).$$

We reiterate the motivation behind this assumption. The optimization of the quantity in (3.2) is an abstraction of the model assumption discussed in the introduction, which hypothesizes that organisms tend to extract the maximum amount of information from their environment with their given sensorimotor equipment. While there are many competing pressures on organisms’ evolution and development, the particular intuition behind this assumption is the following: a biological apparatus will have to maintain as much sensoric information about the current state from the environment as possible, whenever it needs to feed sensory information into a generic and not specialized downstream decision-making and actuation process.

The specific scenario, however, in which we choose, at a specific time step, to extract state information about the beginning of the run is arbitrary. It is in essence only motivated by the simplicity of the interpretation of the resulting memory maps. Other set-ups could easily be conceived. For instance, one could consider maximizing the average information that the memory has at any time about the world state a fixed number of time steps earlier, i.e. an information-optimal delay filter with limited capacity. However, these scenarios are more cumbersome to discuss, and do not contribute substantively to the core argument. It is important to remember that the memory is finite and typically chosen to be small.

Armed with this problem formulation, its search space includes all possible deterministic controller mappings (3.1). To solve this and all following optimization problems, we used an essentially vanilla *simulated annealing* algorithm with some heuristic improvements. However, we emphasize that the solutions are in no way specific to the optimization algorithm and can be performed by any generic optimization tool. In particular, we do not aim to model the details of the process of agent evolution/adaptation enhancing its ability to capture the information about the initial position, but are only interested in the final outcome of such a process. Thus, all following discussions will exclusively deal with the optimized solutions.

## 4. Common concepts

### 4.1. Concepts

Consider an agent with set-up s+ and memory size |M|=8 whose controller is optimized for the agent to capture, as far as possible, the maximal amount of information *I*(*R*_0_; *M*_15_) about its initial position *R*_0_ in a run, where *R*_0_ which is uniformly distributed over $R_{0}={\left\{-5,\;\ldots ,5\right\}}^{2}$.

To interpret this agent, we have to remember that the memory is severely limited, and thus cannot precisely pinpoint the starting position. The best one can hope for is for a given final memory state *m*_15_ to denote a distribution over possible initial positions *R*_0_.

**Figure 3.**
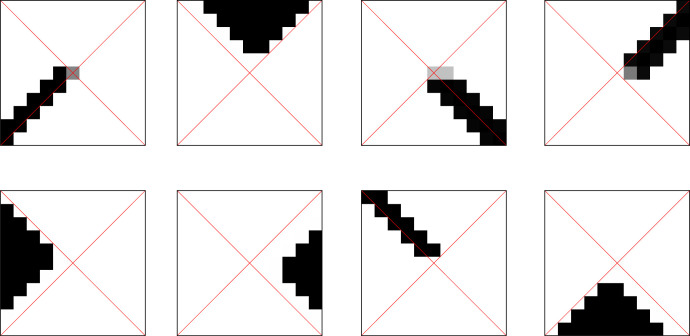
Example solution of initial position capturing.

Concretely, consider figure 3 where each of the eight squares shows in greyscale the conditional probability *p*(*r*_0_|*m*_15_) with *m*_15_ a given final memory state at time step *t* = 15. The diagram shows, for all possible eight final memory states, the distribution of initial states *R*_0_, i.e. the probability that the agent has been initially at position *r*_0_ in the world, when a memory content *m*_15_ = 0, 1, 2, …, 7 is found at time *t* = 15, i.e. the end of the run. Each such distribution over *R*_0_ is represented as an actual square representing all the possible initial positions of the agent in the world.

Note that for each state *m*_15_, we use a separate normalization so, for each separate value of *m*_15_, ${\text{max}}_{r_{0}}p(r_{0}|m_{15})$ will be represented by black and *p*(*r*_0_|*m*_15_) = 0 by white. The agent shown has an utility value of *I*(*R*_0_; *M*_15_) = 2.906 bit. This is very near the limit of $\text{min}[\log|R_{0}|,\log|M|]=3$ bit and thus close to the possible upper bound.

The probabilistic maps of these eight possible memory values *m*_15_ to the initial states can be understood as a *concept* that the agent developed about the world *R*_0_ (or at least its initial state). In particular, each separate value for *m*_15_ carries a certain ‘meaning’ for distributions of starting positions, such as ‘north-triangle’, ‘northeast-diagonal’, ‘east-triangle’, ‘southeast-diagonal’ etc. We call a pair of random variables (*R*, *Y*) (e.g. (*R*_0_, *M*_15_)) which are jointly distributed a *concept* if *Y* is ‘representing’ *R* in some way, i.e. if *I*(*R*; *Y*) > 0. Note that, by abuse of notation, we will reuse the symbol *R* for a generic spatial random variable in the concept (in our examples, typically *R*_0_). Furthermore, we call the values y∈Y *symbols* of the concept.

As mentioned earlier, there will also exist other solutions to the problem to find a good initial position capturer, i.e. one with an equal or similar utility value *I*(*R*_0_; *M*_15_), for example, just an agent where the concepts have been rotated by 90°. This ‘rotation-symmetry’ is an important observation for our discussion and we will return to it later.

At first, we focus on how representative the shown example concepts are and how similar they are to other solutions. We will do this by discussing the possibilities to find a *common* *concept* (*R*, $Y_{*}$) when a set of (not necessarily trivially compatible) *input concepts* {(*R*, *Y*^(1)^), …, (*R*, *Y*^(*n*)^)} are found by different agents.

This concept can be interpreted as a *common* concept of a group of agents in a world *R* that maximizes the collective understanding of external observations in a group of agents. Note that, in the spirit of the philosophy above, we emphatically only model the outcome of optimizing according to an information-theoretical principle. The process of *how* the individuals come to an agreement about the common concept is intentionally not modelled; everything in the following will be independent of the specific algorithm used for the agreement and focuses entirely on the informationally optimal (globally or, sometimes possibly locally) outcome. We will distinguish in the following two possibilities to define such a common concept.

#### 4.1.1. ‘Objective’ common concepts

**Figure 4.**
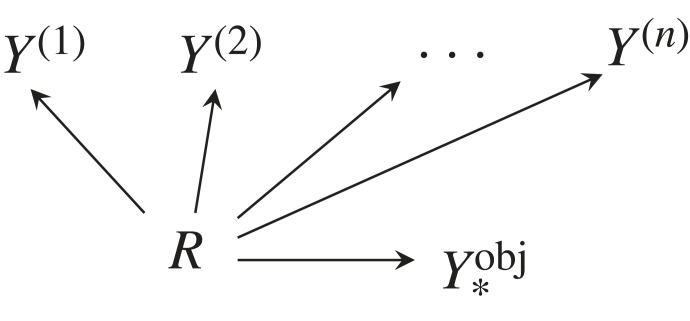
CBN for objective common concept.

Consider the CBN from figure 4. A deterministic mapping $R\to Y_{*}^{\mathrm{obj}}$ which maximizes

4.1 $$\sum_{i}[I(Y_{*}^{\mathrm{obj}};Y^{\left(i\right)})-\alpha \cdot I(R;Y_{*}^{\mathrm{obj}})],$$

defines the *objective common concept $\left(R,Y_{*}^{\mathrm{obj}}\right)$*. The first term $I(Y_{*}^{\mathrm{obj}};Y^{\left(i\right)})$ maximizes the mutual information between the common concept and every input concept, so as to extract as much information as possible from the input concepts.

The term $\alpha \cdot I(R;Y_{*}^{\mathrm{obj}})$ is a bottleneck-type type regularizer with parameter *α* ∈ [0, 1] [13,14]. It counters the trivial solution of just constructing $Y_{*}^{\mathrm{obj}}=Y^{\left(1\right)}\times \ldots \times Y^{\left(n\right)}$ as cross product of all input concepts if the number of states in $Y_{*}^{\mathrm{obj}}$ were sufficiently large, i.e. $\left|Y_{*}^{\mathrm{obj}}|\geq \prod_{i}|Y^{\left(i\right)}\right|$. For our experiments, we set *α* = 0.2. We call this method *objective* because the bottleneck $Y_{*}^{\mathrm{obj}}$ is constructed from explicit knowledge about the world *R*.

#### 4.1.2. ‘Subjective’ common concept

**Figure 5.**
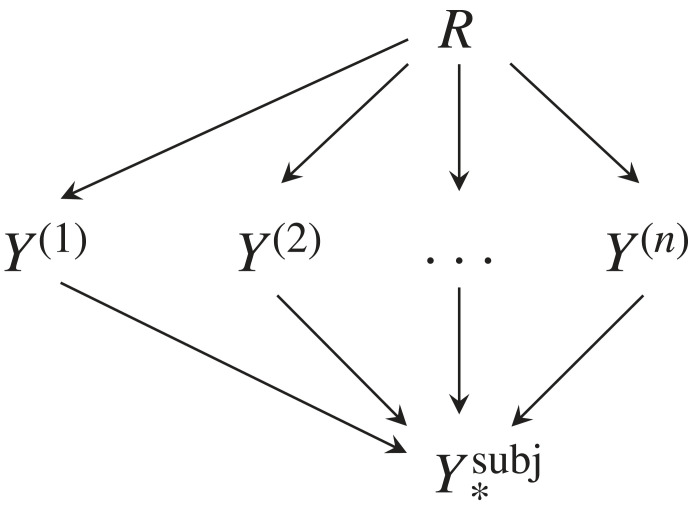
CBN for subjective common concept.

As an alternative, consider the CBN from figure 5. A deterministic mapping $Y^{\left(1\right)}\times \ldots \times Y^{\left(n\right)}\to Y_{*}^{\mathrm{subj}}$ which *minimizes*

4.2 $$I(Y^{\left(1\right)};\ldots ;Y^{\left(n\right)}|Y_{*}^{\mathrm{subj}}),$$

defines the *subjective common concept* $\left(R,Y_{*}^{\mathrm{subj}}\right)$ where we apply the rules for the joint distribution of a CBN. The minimization makes sure that $Y_{*}^{\mathrm{subj}}$ ‘absorbs’ all information common to *Y*^(1)^, …, *Y*^(*n*)^. This method is called subjective because it has only implicit knowledge about *R* and only through the individual input concepts.

### 4.2. Results for the common concepts

**Figure 6.**
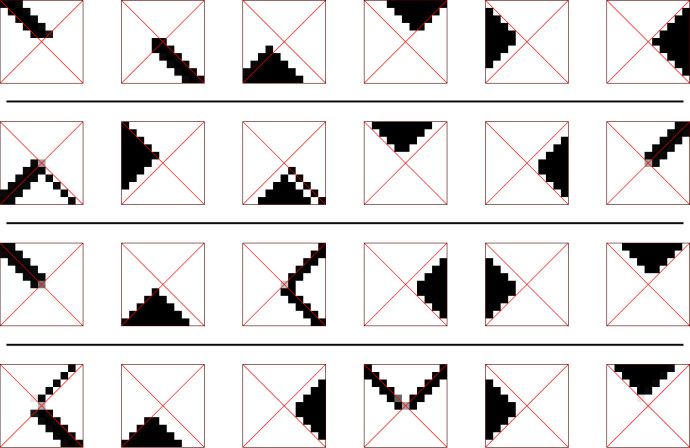
Four input concepts of size |M|=6 for figure 7.

We now consider four agents that were optimized independently to yield the four input concepts shown in figure 6, one concept per row. From these individual concepts, we will now extract both an objective and a subjective common concept. In detail, we see in each of the four rows one concept $\left(R_{0},M_{15}^{\left(i\right)}\right)$, each generated by an initial position-capturing agent with set-up s+ and memory size |M|=6 (hence the six horizontal entries).

**Figure 7.**
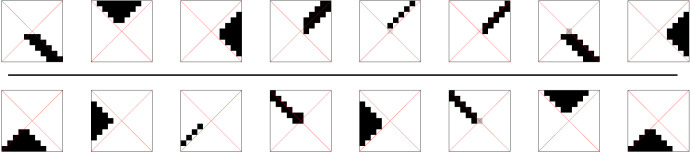
Objective (upper half) and subjective (lower half) common concept.

Using, as before, simulated annealing to optimize the common concept functionals, figure 7 shows both an objective and a subjective common concept (*R*_0_, $M_{*}$) of size $|M_{*}|=8$ each.

One quantity that was hypothesized to potentially show interesting insights was *H*($M_{*}$|*R*_0_), which can be interpreted as ‘superstition’, i.e. information that does not reflect anything in the actual world, but emerges only from the agent concepts themselves. First of all, note that the superstition of the objective common concept is always 0 by construction because $M_{*}$ deterministically depends on *R*_0_. So, it is only for the subjective common concept that it would make sense to consider superstition at all. However, as it turns out, even the superstition of the subjective common concept is only *H*($M_{*}$|*R*_0_) = 0.03 bit which is vanishingly small. Clearly, either the present framework does not tend to favour the emergence of superstition in the above sense, or the scenarios are too simple for such ‘fictitious’ information to emerge in the agent concepts.

The concepts do not fully agree on how to split the space, with different parts of the space being identified by the two concepts. Nonetheless, the information distance between objective and subjective common concept is $D(M_{*}^{\mathrm{obj}},M_{*}^{\mathrm{subj}})=0.38$ bit, which is also quite small.

Because of their pronounced similarity, in the following, we will not continue to calculate both common concepts. We will, from now on, only discuss the objective common concept. Some further objective common concepts are shown in figure 11.

## 5. Symmetry

We observe that all (common) concepts reflect, to a significant degree, the symmetry of the environment. The question that arises now is to which degree can this be captured by the agent collective and whether this symmetry can be characterized via informational criteria.

We will present two methods to measure and analyse these symmetries. Common to both methods is the idea of transforming concepts and comparing them by measuring the mutual information between the transformed concept and its original. Here, it makes sense to distinguish *extrinsic* and *intrinsic* symmetries.

The *extrinsic symmetry* transforms the concept by applying a combination of a rotation, reflection and translation on the external world. It tests if some explicitly known symmetries of the world also translate into the concepts. By contrast, the *intrinsic symmetry* searches for invariants of the world purely from the agent’s perspective. We suggest this as a model for extracting what seems to be a symmetry subjectively for the agent.

These methods are the framework used here for analysing the role of regularities in the agent/world interaction, and especially, what kind of regularities are candidates for being used or exploited by agents in their interaction with the world.

### 5.1. Extrinsic symmetry

An *extrinsic symmetry* operating on a concept (*R*, *Y*) transforms the grid-world $R=Z^{2}$ by applying an *extrinsic symmetry operation* $\xi^{\theta ,\varphi ,x_{0},y_{0}}$, a combination of a rotation *φ* (in 90° steps), a reflection operation *θ*, and a translation by (*x*_0_, *y*_0_)

5.1 $$\xi^{\theta ,\varphi ,x_{0},y_{0}}\;:\;Z^{2}\to Z^{2}$$

and

5.2 $$\xi^{\theta ,\varphi ,x_{0},y_{0}}\;:=\xi_{\mathrm{trans}}^{x_{0},y_{0}}\circ \xi_{\mathrm{rot}}^{\varphi }\circ \xi_{\mathrm{mir}}^{\theta }.$$

The reflection (along the *y*-axis) is described by *θ* ∈ { + 1, − 1}

$$\xi_{\mathrm{mir}}^{+1}(x,y)\;:=(x,y)\;\mathrm{and}\;\xi_{\mathrm{mir}}^{-1}(x,y)\;:=(-x,y),$$

the rotation (in 90° steps anticlockwise) by φ ∈ {0°, 90°, 180°, 270°}

$$\xi_{\mathrm{rot}}^{0^{\circ }}(x,y)\;:=(x,y)\;\xi_{\mathrm{rot}}^{90^{\circ }}(x,y)\;:=(-y,x)$$

and

$$\xi_{\mathrm{rot}}^{180^{\circ }}(x,y)\;:=(-x,-y)\;\xi_{\mathrm{rot}}^{270^{\circ }}(x,y)\;:=(y,-x)$$

and finally, the translation by $(x_{0},y_{0})\in Z^{2}$

$$\xi_{\mathrm{trans}}^{x_{0},y_{0}}(x,y)\;:=(x+x_{0},y+y_{0}).$$

**Figure 8.**
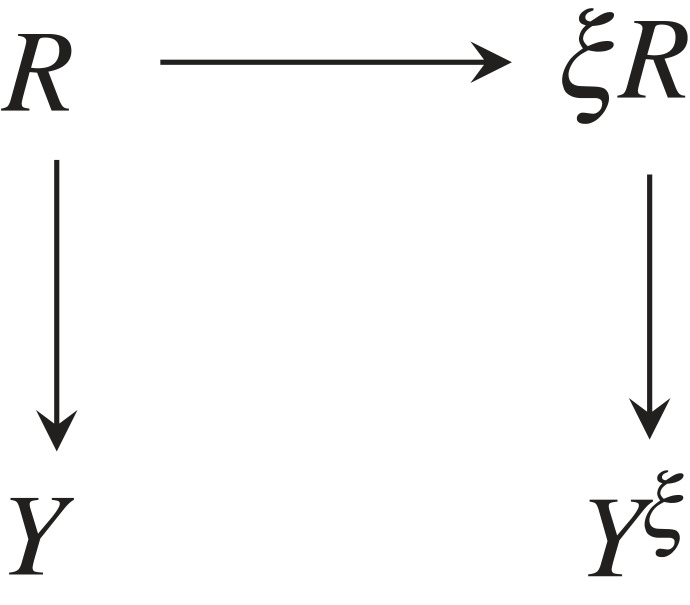
CBN for calculating the utility of the concepts under an extrinsic symmetry. The mutual information between *Y* and $Y^{\xi }$ can be considered as a measure for the degree of equivariance for the concept used (the vertical arrows).

The application of the operation $\xi^{\varphi ,\theta ,x_{0},y_{0}}$ transforms the world *R* and gives us two new probabilistic mappings *R* → *ξR* and *ξR* → *Y*^*ξ*^ (figure 8) where we omit the parameters for *ξ* for clarity.

The first mapping applies the operation $\xi^{\varphi ,\theta ,x_{0},y_{0}}$ on *R* via

5.3 $$\;\mathrm{Pr}(\xi R=r^{\prime }|R=r)\;:=\delta_{r^{\prime },\;\xi (r)}$$

5.4 $$=\left\{ \begin{array}{cc} 1\; & \mathrm{if}\;r^{\prime }=\xi (r)\;\\ 0\; & \mathrm{else}.\;\; \end{array}\right.$$

It corresponds to a deterministic mapping of each *r* to *ξr* ≡ *ξ*(*r*).

The second mapping *ξR* → *Y*^*ξ*^ is chosen as a ‘copy’ of *R* → *Y*:

5.5 $$\mathrm{Pr}(Y^{\xi }=y|\xi R=r)\;:=\mathrm{Pr}(Y=y|R=r).$$

The structure of this mapping can be seen in figure 8.

We now define the *utility for an extrinsic symmetry operation* $\xi^{\varphi ,\theta ,x_{0},y_{0}}$ for the concept (*R*, *Y*) via

5.6 $$I(Y;Y^{\xi }),$$

where a higher value indicates that the symmetry *ξ* is better respected by the concept. Stated in the language of group theory, this is a measure of the degree of *equivariance* realized by the concept. When this quantity is maximal, it means that the transformed concept can be used equivalently to the original. Crucial for this interpretation is the fact that the rightmost vertical arrow has *not* been transformed together with the variables but is a clone of the leftmost vertical arrow.

For the term in (5.6) to be maximal, for each selected *y*, one will have a distribution of *p*(*r*|*y*), which gives under transformation *ξ* a distribution *p*(*ξr*|*y*). Each of the outcomes in the support of the latter is mapped by the given concept via *p*(*y*′|*ξr*), and the maximization of the information demands that, since *y* was chosen deterministically, that the *y*′ must also be deterministically given and will depend only on the original *y* and on *ξ*, and hence will define a specific $y^{\xi }$. Whenever this is not the case, the concept does not respect the symmetry. When (5.6) is maximized for all symmetry operations, all these concepts are informationally equivalent. In this case, the concepts can be seen as forming a ‘gauge theory’.

However, the symmetries do not have to be strictly preserved. The measure in (5.6) allows us to measure to which extent this is actually the case. In our concrete case, this utility measures how much a rotated/reflected/translated concept has in common with the original one. Note that, because of the use of information theory, possible symbol permutations are ignored. Note, finally, that with this method, we are also able to interpret a sensor mapping *R*_*t*_ → *S*_*t*_ itself as a concept and calculate its symmetries.

### 5.2. Intrinsic symmetry

The extrinsic symmetry concept assumes a known external world symmetry operation. However, it is interesting to consider the situation when no such external model is available, and all operations need to be considered in relation to the agent, more specifically, in relation to its embodiment.

For this purpose, we introduce a formalism to ‘reinterpret’ the symbols of sensors and actuators by interposing a permutation symmetry operation between the impinging sensor signal and the one that the agent actually receives, and similarly, the actions are reinterpreted by a permutation before being sent out to the actuator. Note also that the permutation operators for the sensors and for the actuators are not coupled, but can be separately chosen.

**Figure 9.**
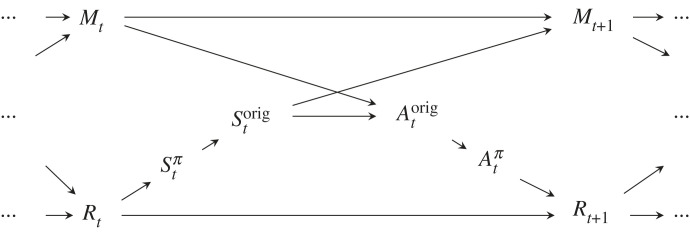
Permuted embodiment for the perception–action loop as a CBN.

Formally, we define a *permuted embodiment* for an agent as in figure 9. In comparison to figure 1, the original sensor *S* is replaced by *S*^*π*^ → *S*^orig^ and the original actuator *A* by *A*^orig^ → *A*^*π*^, where *π* = (*π*_*S*_, *π*_*A*_) denotes a pair of (deterministic) permutation maps for sensors and for actuators

5.7 $$\pi_{S}\;:\;S^{\pi }\to S^{\mathrm{orig}}$$

and

5.8 $$\pi_{A}\;:\;A^{\mathrm{orig}}\to A^{\pi }.$$

The operations *π* = (*π*_*S*_, *π*_*A*_) on the sensorimotor layer form a group. We say that *π* defines an *intrinsic symmetry operation*.

This operation introduces a symmetry operation that does not act globally on the state (which is not something an agent may be able to model intrinsically) but at a level internal to the agent and which would be thus—in principle—accessible to the agent. This permits us to study various alterations of the sensorimotor interaction with the environment, while keeping the interface between controller and sensor/actuation level on the one hand and the interface between the sensor/actuation level and the environment unchanged. All alterations happen in the intermediate boundary layer.

To evaluate an intrinsic symmetry operation (*π*_*S*_, *π*_*A*_) on an initial position-capturing agent, we first generate a sample of *n* concepts $\left\{(R_{0},M_{15}^{\left(1\right)}),\ldots ,(R_{0},M_{15}^{\left(n\right)})\right\}$ by repeatedly optimizing initial position-capturing agents with an equivalent set-up. Because the optimization does not have a unique optimum, one in general obtains a variety of resulting concepts. At this stage, both the evaluated agent and the other agents have not yet incorporated the modifications from figure 9. From this set of concepts, we compute an objective common concept $\left(R_{0},M_{*}^{\mathrm{obj}}\right)$.

Given the specific intrinsic symmetry operation (*π*_*S*_, *π*_*A*_) we wish to evaluate, we apply it on the thus achieved perception–action loop without changing the controller, i.e. for this evaluation, we do not optimize the utility from (3.2) again. Instead, we calculate the concept that results when applying the permutation $\left(R_{0},M_{15}^{\pi }\right)$, and define the quality of this permutation as

5.9 $$I(M_{*}^{\mathrm{obj}};M_{15}^{\pi }).$$

A higher value indicates a higher degree of (intrinsic) symmetry. Informally spoken, we measure to which degree the permuted concept conforms to the common concept of the sample. Permutations that achieve a high value indicate that they permit the permuted concept to be a substitution for the original one and thus that this permutation identifies a suitable equivalence of concepts.

### 5.3. Results for extrinsic and intrinsic symmetries

Figure 11 shows a comparison of the relevant results. The upper half of the figure is for set-up s+, the lower half for the more complex set-up sq.

**Figure 10.**
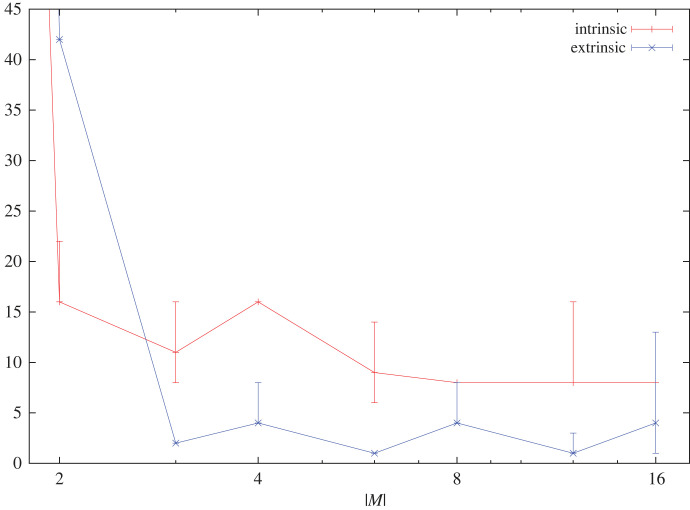
Number of good intrinsic and extrinsic symmetries depending on memory size.

#### 5.3.1. Extrinsic symmetry of the set-up

**Figure 11.**
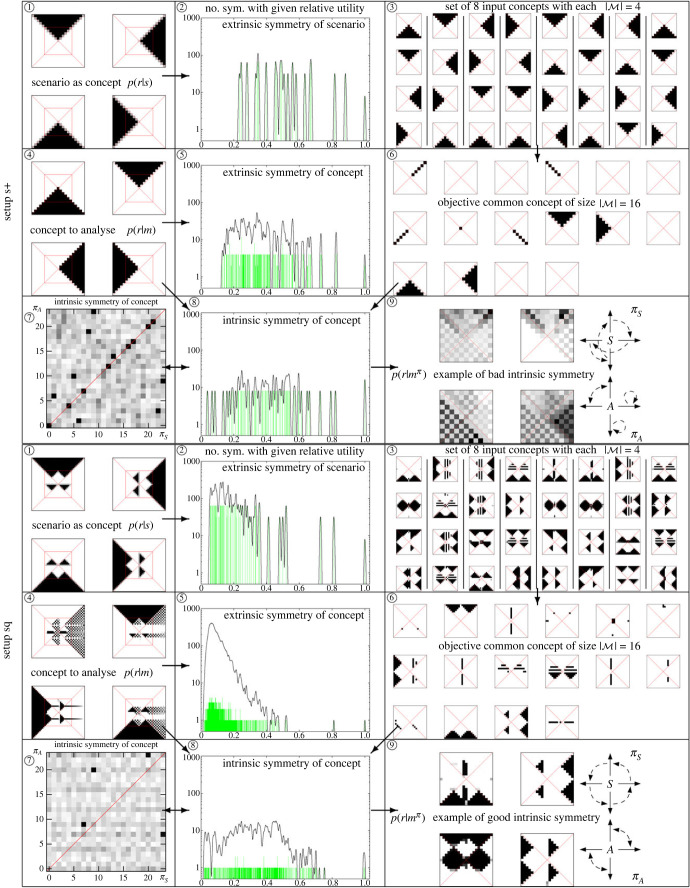
Overview diagram: ①④ concepts, ②④⑧ symmetry counts, ③ individual, ⑥ common concepts; ⑦⑧ intrinsic symmetries—see text for details.

We begin by showing extrinsic symmetries in figure 11, subfigure ① and ②. Specifically, subfigure ① shows the set-up used as map of possible sensor outcomes (interpreted as concepts) *p*(*r*|*s*). As with memory-based concepts, the marked box inside of each symbol denotes the probability of locations belonging to *R*_0_, given the different sensor inputs *s*. This region visualizes the areas of the concept that will be compared using the mutual information when the concept is transformed.

We note that we use $R_{0}={\left\{-5,\ldots ,5\right\}}^{2}$ for set-up s+ and $R_{0}={\left\{-10,\ldots ,10\right\}}^{2}$ for set-up sq.

Of the translation operations, only those that map *R*_0_ on a subset of the shown positions in the concept are tested, i.e. only translations with $\text{max}(|x_{0}|,|y_{0}|)\leq 5$ for set-up s+ or ≤10 for set-up sq. The corresponding extrinsic *symmetry spectrum* in ② shows on the *y*-axis of the histogram how many symmetries one finds with a given value of $x=\frac{I(R;Y^{\mathrm{transformed}})}{\text{max }I(R;Y^{\mathrm{transformed}})},$ with a high value of *x* being a good match. Note the logarithmic scale for the *y*-axis.

To make the peaks more visible, we slightly smoothed the histogram. The rightmost peak comprises the eight dihedral (perfect) symmetries with no translation (*x*_0_ = *y*_0_ = 0). The next best peak (second rightmost) covers translations of length 1. We find that the peaks are mostly ordered by their translation distance $\sqrt{x_{0}^{2}+y_{0}^{2}}$. An analogous analysis can be made for the set-up sq.

#### 5.3.2. Extrinsic symmetry of the concepts

We now consider the extrinsic symmetries of the concepts. These are shown in figure 11, subfigures ④ and ⑤. Subfigure ④ shows a concept derived from an initial position-capturing agent (*R*_0_, *M*_15_) with |M|=4 and its extrinsic symmetry spectrum is quite similar to the set-ups in ① and ②. Note the subtle difference in allocating the diagonals of the space. While the sensors on the diagonal are randomly assigned to one or the other possible directions, the memory-based concepts are deterministic due to their model.

Unsurprisingly, also here we find that the peaks are mainly ordered by translation distance. For set-up s+, we now have a peak with the best four operations; this peak encompasses the reflection with respect to the *x* resp. *y* axis. If we reflect along the *x*-axis in the state space, additionally, we have to translate the concept by 1 in the *y*-direction to get it to match perfectly with the original one.

For the concepts emerging in set-up sq, we see that there is only one best symmetry, the identity. The next best symmetry, a reflection along the *x*-axis, is much weaker.

#### 5.3.3. Intrinsic symmetries

We now consider an objective common concept (*R*_0_, $M_{*}$) which is used to evaluate intrinsic symmetries. It is derived from eight other solutions to capture the initial position (shown in figure 11, subfigure ③: each column of four symbols forms one input concept). The common concept is shown in subfigure ⑥. It has been computed for a theoretically available memory size of $|M_{*}|=16$ symbols; however, it turns out that when it is computed, only nine symbols are actually populated.

#### 5.3.4. Intrinsic symmetry of a concept

For the concept in subfigure ④, the spectrum of the intrinsic symmetry is shown in subfigure ⑧. Diagram ⑦ also shows these intrinsic symmetries but in a different way. The *x*-axis resp. *y*-axis enumerate the different possibilities for the permutations for *π*_*S*_ and *π*_*A*_ with 0 standing for the identity. The grey values represent the symmetry ‘strength’ measured via $I(R;M_{15}^{\pi })/{\text{max}}_{\pi }I(R;M_{15}^{\pi })$. We enhance contrast for values that are close to 1, i.e. to being represented by black. The diagonal in this map is specifically constructed to encompass ‘synchronized’ embodiment permutations where permutations of actions and sensors are matched up correspondingly, i.e. *π*_*S*_ = *π*_*A*_. Of course, the introduction of such synchronized permutations is only well-defined if, as in our case, sensor and actuator values can be associated and ordered in precisely the same way.

Subfigure ⑨ shows one of the best (for set-up sq) resp. worst (set-up s+) concepts $\left(R_{0};M_{15}^{\pi }\right)$ after applying an intrinsic symmetry operation (*π*_*S*_, *π*_*A*_) as a representative example of how such operations will look. This operation has two permutations, which are shown to the right of the concept in the respective subdiagram. The four possible values for *S* resp. *A* are shown as solid arrows and their permutation mappings with dashed arrows. In case of set-up s+, the 16 best symmetries are similar to the eight rotations and reflections of the rightmost two input concepts shown in ③. In case of set-up sq, the four best symmetries are similar to the example shown, but additionally reflected along the *x*- and/or *y*-axis.

#### 5.3.5. Symmetry dependence on memory size

The symmetry depends to some extent on the size of the memory |M|. This dependence is shown in figure 10. It shows the number (*y*-axis) of good extrinsic resp. intrinsic symmetries (with ‘good’ denoting symmetries that achieve at least 85% of the maximal symmetry utility) for an initial position-capturing agent with set-up s+ according to memory size |M| (*x*-axis). The error bars show the number of symmetries achieving at least 82.5% resp. 87.5% of the maximal symmetry utility.

## 6. Discussion

We considered agents embedded in a simple world, and modelling the agent’s perception of the world as concepts that translate certain features of the world (the agent’s initial positions) into internal memory states of the agents. Based on these concepts, we studied how individual concepts could be ‘merged’ into collective concepts. These collective concepts are more expected to extract ‘objective’ features of the world, as they do not just depend on the serendipitous concept of a single agent. Against these collective concepts, we investigated symmetries; these were either extrinsic symmetries of the given world or intrinsic symmetries that—in principle—could be explored by the agents themselves and do not rely on a birds eye view-type prior knowledge of externally given symmetries.

When generating the common concepts, both objective and subjective methods end up with almost similar concepts in our scenarios if, as in our case, the input concepts are mostly deterministic. So one can save computation resources by calculating only the objective one which requires only the consideration of pairwise correlated memory variables rather than the joint distribution over the complete set of memory variables.

Both methods deal with the phenomenon that, for some locations in the world, the agents disagree on how to group them to given memory symbols (in our example particularly prominent for e.g. the four diagonals in set-up s+). In addition to assigning the ‘original’ symbols for undisputed areas, the common concept methods are able to identify the disputed areas and assign new symbols to them. If we enlarge the memory size for the individual agents, they would find some of these new symbols as well. In our example, an agent with a larger memory size would also find the diagonals but with much lower accuracy.

However, it turns out that forcing the agents to converge on common concepts produces novel concepts not discoverable by individual agents. To illustrate, consider the symbol for the ‘centre of the world’ (figure 11 for set-up s+, subfigure ⑥). A symbol for ‘centre of the world’ was never found by an individual agent in any of our experiments. Thus, a new, hitherto unseen, symbol class emerged by considering a whole group of agents instead of individuals. Not only that, but the common concepts usually capture significantly more symmetry than the individuals that exhibit substantially stronger deviations from the symmetries.

We also found that ‘good’ agents’ concepts and especially common concepts tend to exhibit a higher degree of ‘symmetry’. We considered two methods to study the quality of the given symmetries.

With the extrinsic symmetry method, rotation and reflection symmetries were found but the translations did not stand out. This is also partly reflecting the symmetry properties of the gradient field. As expected, small translations were not completely asymmetrical. In general, however, the degree of symmetry was vaguely ordered by its translation distance.

As opposed to the extrinsic ones, the intrinsic symmetry just observes which changes in the fixed patterns of the agent’s interaction with the environment (i.e. which actuator/sensor permutations) have no (bad) effect on its concept. This method is additionally stabilized by the fact that we do not compare a permuted concept with the original individual concept but with a common concept derived from multiple agents. This common concept is free of idiosyncratic decisions of individual agents and provides a more universal representation for a task than any individual solution.

Intrinsic symmetries may sometimes incorporate certain extrinsic symmetries (rotation, reflection) but they include many more operations. Searching for high-symmetry intrinsic operations corresponds to forcing the given agent to conform to the common concept without optimizing their controllers again by, in a way, ‘transplanting their brain into a rewiring of their sensorimotor embodiment’. Searching for the best intrinsic operation that best conforms to the common concept can be seen as partly a re-optimization of the controller, but only as far as the sensory input and actuator output are concerned, rather than the controller itself. This has the consequence that, in searching the intrinsic symmetry space, in total, in the example shown, we only test a vanishingly small (3.1 × 10^−17^-th) proportion of the total search space for possible controllers.

This indicates a more remarkable phenomenon: the common concepts, which, as mentioned, represent higher symmetries than the individual can extract, and also possibly new symbols that the individuals do not achieve (‘centre of the world’, see above) can be ‘retrained’ into the individuals, here by searching through the space of intrinsic permutations, which is far smaller than a full adaptation of the controller. In short, individuals produce concepts that are consolidated in the collective, and the consolidated, and more high-quality common concepts can now, in turn be re-acquired by the individuals. This indicates, still on a very abstract level, a potential route for the emergence of structured joint communication from individuals, which then again influences the individuals, in turn. While the model is too minimalistic to be able to talk about a language, it highlights possible routes for the mutual influence of individual concept formation and collectivistic communication. One may speculate that an iterative application of individual concept formation and common concept extraction might produce variants of more structured concepts. If and how such concepts could be accumulated over time remains a question to be studied in the future.

As a final observation, we see that by increasing the agent’s memory size the number of best extrinsic symmetries drops to 1. The latter means that identity becomes the only remaining symmetry operation for large agent memories. However, for intrinsic symmetries, this is different. Rather, it turns out that intrinsic symmetries are not too sensitive to variations of the concept due to symmetry operations. This raises another possibility: the intrinsic symmetries may provide a hint as to how to choose an optimal memory size of an agent. With growing memory size, the agents begin to detect that not every symmetry they can probe in principle corresponds to an actual symmetry in the world. For intrinsic symmetries, the drop stops at approximately eight operations, and that also indicates a natural choice for an agent's memory size in the considered set-up.

Research into automatic discovery of symmetry and structure has made significant strides in recent years, with particular advantages in continuous systems, which may exhibit physics-mediated symmetries and constraints (see e.g. [15]). The concept of discovering such constraints also has, beyond application in physics, ramifications for understanding biological regulation [16]. The intrinsic recovery of the structure of the environment through embodiment [17,18] has received an increasing amount of attention. The present approach shares some aspects with these, but adds a few more. First of all, due to its discreteness, the space can be assumed as far more unstructured than a smooth embedding; much work is to be done to scale the approach up to larger discrete systems. Furthermore, any symmetry is characterized by ‘agreement’ across the collective common concepts. It is not any longer necessarily an objective symmetry, but something that has been jointly agreed upon by a group of agents. In other words, concepts and potential structures emerge through agreement, with the potential to have some group-subjective structure.

The importance of agent groups to construct common frameworks has come increasingly into focus and tools developed in the context of the active inference framework might provide ways to address the complexities in larger scenarios. Of special relevance is the growing awareness concerning this class of models, namely that intelligent inference might require, as an obligatory feature, a partitioning of the system into subagents which carry out inference individually, and then build up a joint representation [19]. In this view, the collective of agents is thus not an exception, but the rule of how models emerge, and shared models are at the core of such a perspective. Related to the concept of superstition studied above (which turned out to be of no great import in the present minimal model) is the phenomenon of echo chambers, where agents create their own epistemic communities, which can detach themselves from an external reality to amplify their own confirmation biases [20].

The approach we espoused here was inherently information-theoretical. This has a number of consequences: deviations from perfect symmetry are no longer measured in terms of (possibly arbitrarily chosen, typically Euclidean-based) distance costs, but in terms of informational deviation, which implicitly acts as a ‘complexity’ cost for an agent to have to deal with the surprise of an invariant not being maintained.

This suggests a pathway to a more principled understanding of why and when symmetries can be expected to be found and to be useful for a decision-making agent. Also, it might indicate more fundamental reasons why, apart from aesthetical and other externally imposed constraints, human observers and decision-makers might seek out symmetries, namely for reasons of information parsimony.

If some type of information parsimony would indeed turn out to be one of the drivers to prefer more symmetric descriptions of the world, it might be traded off in some situations against other, less symmetric, but otherwise more parsimonious descriptions. In future, this might provide a prediction about the landscape of trade-offs between structurally compact controllers or world descriptions versus practically compact, but less well-structured models and a more systematic approach to characterize when aesthetically attractive solutions are preferrable and when they have to give way to pragmatic, but less attractive models.

## 7. Conclusion and outlook

We discussed two techniques to generate a common perspective by conflating the individual perspectives of a group of agents. Through this common perspective, we were able to analyse the similarity of individual agent representations and find common classifications of the environment. Additionally, some features of the world are only (or at least much more easily) detectable in the common perspective. We did not model the process of agreeing between these agents and only used very general information-theoretical principles which make them applicable to other scenarios as well.

We found evidence that good classifications of the environment capture many of its symmetries. While individual concepts may suffer some symmetry breaking, common concepts will reveal these symmetries. To analyse these symmetries, we developed two information-theoretical approaches. In the extrinsic approach, we measure for every symmetry transformation of the environment the degree to which the concept is respected. This approach abstracts away from how we achieved the classification. By contrast, the intrinsic approach is only suitable for agents interacting with an environment through a perception–action loop. Here, we analyse which modifications of the embodiment lead to agents who are ‘similar’ to the original one. Since we measure this similarity indirectly by comparing the transformed concept to a common concept, a symmetry broken by an individual’s concept does not affect this method. The intrinsic method provides insight into the agent and its perspective on the environment. It identifies symmetries beyond the geometrical symmetries of the world found in the extrinsic case. The intrinsic symmetries correspond to changes of the agent’s embodiment, which cannot be detected by the agent. In the future, considering such intrinsic symmetries could also help to extract non-obvious structural regularities in the environment by the agent.

## Data Availability

This article has no additional data.

## Endnote

1 *t* = 15 is an arbitrary choice for our experiments, other choices lead to similar results.
